# A thematic network for factors affecting the choice of specialty education by medical students: a scoping study in low-and middle-income countries

**DOI:** 10.1186/s12909-021-02539-5

**Published:** 2021-02-10

**Authors:** Yaser Sarikhani, Sulmaz Ghahramani, Mohsen Bayati, Farhad Lotfi, Peivand Bastani

**Affiliations:** 1 Student Research Committee, Shiraz University of Medical Sciences, Shiraz, Iran; 2 Health Policy Research Center, Institute of Health, Shiraz University of Medical Sciences, Shiraz, Iran; 3 Health Human Resources Research Center, School of Management and Information Sciences, Shiraz University of Medical Sciences, Almas Building, Alley 29, Qasrodasht Ave, Shiraz, Iran

**Keywords:** Specialty selection, Influential factors, Low-and middle-income countries, Medical students

## Abstract

**Background:**

Medical specialty selection is a complex phenomenon that can affect the performance of health systems, community health, and physicians’ lives. It is essential to identify the key factors influencing the choice of specialty for evidence-based policymaking. This scoping review aimed to provide a comprehensive map of evidence regarding the factors influencing the choice of specialty by medical students (MS) in low-and middle-income countries (LMICs) and also to determine knowledge gaps.

**Methods:**

We carried out a systematic search on six online databases from January 2000 to May 2020. We used a five-step scoping review method proposed by Arksey and O’Malley. We synthesized the data using a quantitative content analysis approach. Then, we developed a thematic network as a conceptual map for a better understanding of the concept.

**Results:**

The analysis led to the development of five main themes, including personal determinants, life fulfillment aspects, influential career aspects, educational determinants, and interpersonal effects. Moreover, the most frequent sub-themes were specific personal factors, controllable lifestyle, quality of working life, and future working conditions.

**Conclusion:**

This review provided evidence on the factors influencing the choice of specialties. In order to support physician workforce policy with more precise evidence, it is necessary to explore the weight and ranking of these factors based on the socioeconomic contexts of the countries. This study also indicated that factors such as ethical values, various aspects of medical philosophy, and immigration tendencies are areas for further investigations.

**Supplementary Information:**

The online version contains supplementary material available at 10.1186/s12909-021-02539-5.

## Background

Selection of medical specialty is an important issue in health human resource planning that has received increasing attention in recent years [1]. The tendency toward the selection of different medical specialties can largely determine the future landscape of the physician workforce in a healthcare system [2]. Failure to adopt appropriate strategies to adjust the selection of medical specialties may have some undesirable consequences for health systems. The imbalance between various specialties is one of the significant adverse outcomes [3, 4]. As a result, this problem leads to other complications such as shortage of physician in some specialties and particularly primary health care [5], physicians’ unequal geographical distribution [3, 4], a mismatch between the real needs of the community and existing health workforce, increased healthcare costs due to the over-specialization of health services [6], and limited access to health care particularly in remote and rural areas [7]. It has been widely discussed that the shortage of some medical specialties, as well as the unequal distribution of physicians, are two worldwide healthcare problems. However these conditions are worse in low-and middle-income countries (LMICs) [8].

Choosing a medical specialty is a complex process that has many factors involved [9]. Studies have indicated that financial factors [6, 10–12], personal interests [10, 13], personality type [14–16], academic and educational determinants [17–19], demographic characteristics [13, 16, 20], as well as cultural and social values [5, 16, 21] are among the leading factors that predict the choice of medical specialty as a career. The driving forces behind the choice of medical specialty depend to some extent on the cultural and socioeconomic conditions of the countries [5, 22]. A review study showed that attitude toward community problems, interest in voluntary work, length of residency training, family influence, awareness of rural needs, and intellectual challenge are factors associated with choosing primary health care as a specialty career [8]. However, based on our searches, no comprehensive study has reviewed the factors influencing the choice of medical specialty in LMICs. Although there is only one relevant study by Puertas et al. [8], they have focused solely on primary care specialties, which can affect the comprehensiveness of the evidence.

Because medical specialty choices can affect the performance of health systems, community health, as well as physicians’ personal lives and career paths, identifying the determinants of these choices is of great importance [23]. Understanding the leading factors associated with the selection of medical specialties is essential for evidence-informed policymaking, better medical education planning [6, 7, 9, 24, 25], more balanced distribution of health human resources [1], enhancing physicians’ career planning, improving doctors’ performance, as well as accommodating the selection of medical specialties with the preferences of physicians and the needs of the community [26]. Due to the diversity of studies in this area, a review is necessary to combine the available evidence. Therefore, in order to provide a comprehensive map of the evidence and to determine the research gaps, this scoping review aimed to explore the main factors influencing the choice of specialty by medical students (MS) in LMICs.

## Methods

This scoping study aimed to explore and map the evidence on the main factors influencing specialty selection by MS in LMICs. We categorized countries based on the classification and data provided by World Bank. According to the Word Bank 2019, countries with a Gross National Income (GNI) of less than $12,536 are categorized as LMICs [27]. We used a scoping review method because it allows the inclusion of studies with diverse samples and designs [28]. This type of review also makes it possible to identify main factors associated with a concept, to map out evidence on a topic, and to specify research gaps in an area [29]. Accordingly, we used the scoping review method proposed by Arksey and O’Malley [28]. This approach includes five separate steps: 1- Determining the research question, 2- Finding related studies, 3- Selecting relevant studies, 4- Extracting and charting the data, as well as 5- Collating, summarizing and reporting the findings.

### Determining the research question

Although the research question determines the scope of a study, a scoping review is carried out in an iterative process. Therefore, in this study the research question developed progressively during the review. In this study, identification of leading factors associated with medical specialty selection in LMICs was considered as the outcome of interest. Moreover, MS with the clinical experiences was the second component of the research question. Consequently, the aim of this study was to answer this question: ‘what are the main factors associated with the selection of specialty by MS in LMICs?’

### Finding relevant studies

Before conducting a comprehensive review, we searched several database to ensure no similar study was available. We carried out a systematic search on six online databases, including Scopus, PubMed, ProQuest, Embase, Web of Science, and ScienceDirect, in order to find relevant studies published from January 2000 to May 2020. We also searched many search engines and websites for other types of reports. First, we determined three categories of search terms by performing a preliminary literature review. Then, we amended and completed the categories during the review process. We merged the search terms in each group applying logical operator ‘OR’, and then we combined the categories using logical operator ‘AND’. Table 1 shows the search strategy for the study. Initially, we retrieved 10,004 studies using this search strategy. After deleting duplicates, 3883 articles were entered into the evaluation phase. We used EndNote X7.1 as the reference management software.

**Table 1:** Search strategy for factors influencing the choice of specialty by medical students in LMICs

Searched databases	PubMed, Scopus, Web of Science, ProQuest, ScienceDirect, Embase
**Search strategy**	**#1 AND #2 AND #3**
**#1**	“Medical Student” OR “Interns” OR “Graduating students” OR “Graduating medical students” OR “Junior physician” OR “House staff” OR “Medical internship”
**#2**	Specialty OR Medical specialty” OR “Specialty Choice” OR “Specialty selection” OR Career OR “Choice of specialty”
**#3**	Factor OR Preference OR Determinant OR Predictor OR Motives OR “Driving factor”
**Limitations**	Language: Articles with full-text in English Time: January 2000 – May 2020 Article type: Original researches and discussion papers

### Selecting relevant studies

We conducted a three-step iterative peer review process to select the studies relevant to the aim of the study. At each step, we refined our search strategy, searched for new studies, and reviewed them. In all three steps, the evaluation was performed by two reviewers (YS and PB) independently. In order to become more familiar with all steps of the study, the two reviewers carried out a pilot project before the main study. We used the three criteria of scoping review studies to develop the key question of the study as well as at all evaluation stages. In this regard, MS with clinical experiences, factors associated with the selection medical of specialty, and LMICs were considered as “Population, Concept, and Context” (PCC) respectively.

Firstly, we screened titles of the papers based on the study question, and 342 articles were accepted for further evaluation. After removing irrelevant titles, abstracts of the remaining papers were evaluated, and those did not meet the objective of the review were deleted. Accordingly, we selected 124 full-text articles for further evaluation. Finally, we apprised the full-text papers and 29 studies [17, 23, 30–56] were selected for final analysis. We evaluated the quality of the full-text articles using the “*Strengthening the Reporting of Observational Studies in Epidemiology*” (STROBE) checklists [57] and the “*Critical Appraisal Skills Programme*” (CASP) tools [58]. At all steps of the evaluation phase, a third researcher (MB) reviewed cases of disagreement for final inclusion.

### Inclusion/exclusion criteria

**Fig. 1:**
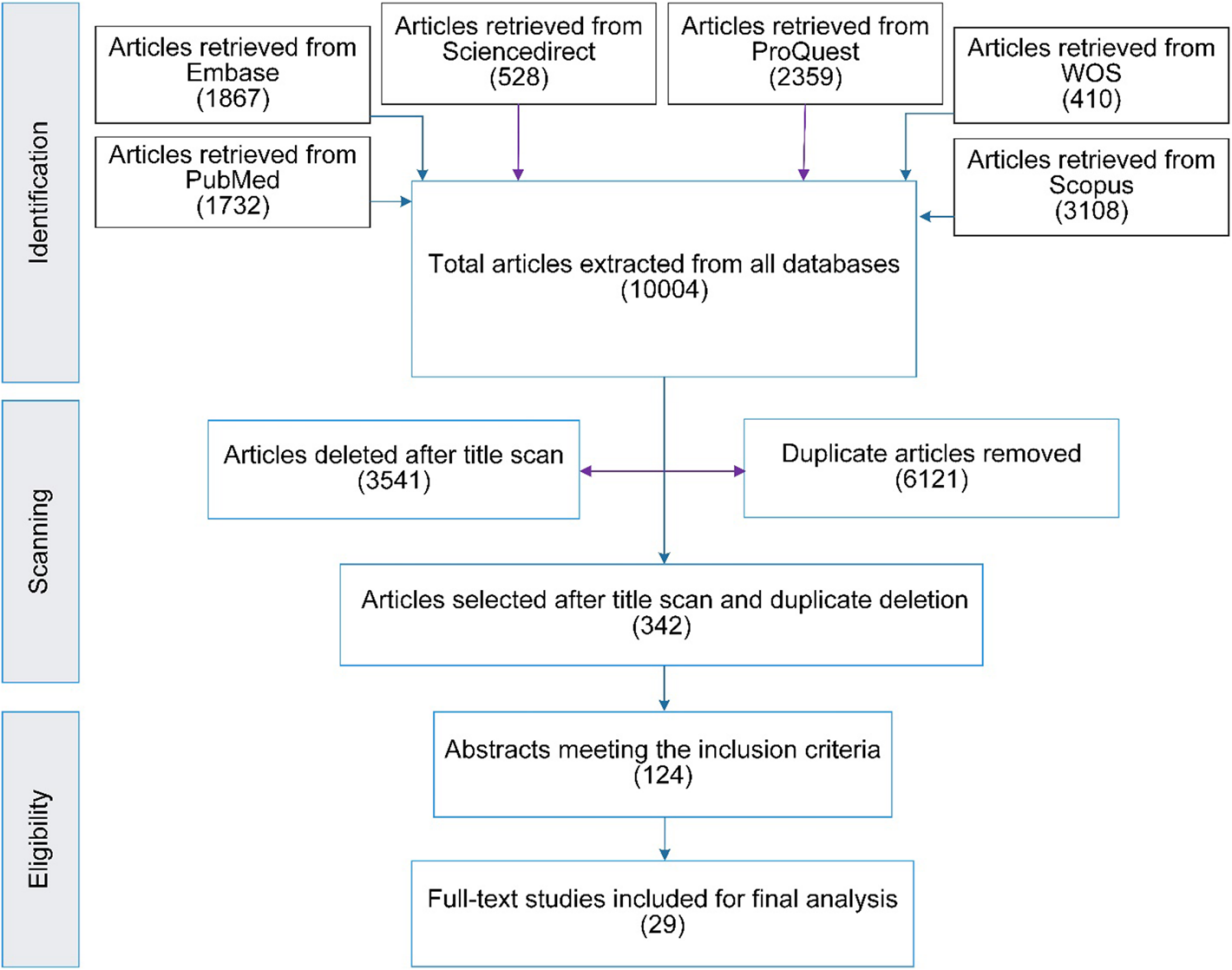
PRISMA Flowchart of the included papers in the scoping review

Because of language and translation restrictions, we only selected studies with full-text in English. Because individuals’ preferences changes along with socioeconomic conditions and in order to explore the latest changes in the topic, articles published after 2000 were selected. Only original articles and discussion papers were included. However, we excluded letters, commentaries, and review studies. Because preferences of MS change during the education period, we selected studies focused on MS with clinical experiences. Also, to provide a more general view of the concept, we excluded those articles that focused on a specific career. The paper selection process is presented in Fig. 1 in the form of a PRISMA flowchart.

### Charting the data

In order to extract relevant data from the selected articles, we developed a data-charting form. In this regard, three researchers (YS, PB, and MB) carried out the charting jointly through an iterative process, so that they extracted data and updated the data-charting form continuously (Appendix 1).

### Collating and summarizing the data

According to Arksey and O’Malley’s guideline for scoping review, we used thematic content analysis to synthesize the data [28]. In this regard, we applied Graneheim and Lundman’s approach in six steps, including becoming familiarized with the data, initial coding, searching for themes, reviewing themes, defining the themes, and reporting the results [59]. Two researchers (SG and FL) analyzed the data independently, and then they compared the results to confirm similar findings and achieve agreement on discrepancies. At the first step of thematic analysis, the researchers became immersed in the data through multiple readings of the full-text articles. Then they determined initial codes based on the objective of the study. In the third step, the researchers performed an interpretive analysis of the preliminary codes to identify main themes and their related sub-themes. The next step was the revision of the themes. To this end, the research team held a joint discussion meeting to refine, merge, separate, or delete primary themes if it was necessary. Lastly, the research team named and labeled the themes and sub-themes in terms of relevancy (Appendix 2). The main themes and the sub-themes are demonstrated in the form of a table. Also, for a better understanding of the breadth of evidence, the table includes frequency of studies related to the development of each theme. Eventually, we prepared a thematic network of the evidence as a conceptual map to provide a more comprehensive perspective into the factors associated with the selection specialty by MS.

To enhance the trustworthiness and rigor of the content analysis, we used the proposed criteria of Guba and Lincoln, including credibility, confirmability, dependability, and transferability [60]. To increase the credibility of the findings, we used prolonged engagement with the content and performed peer-check during data analysis. We tried to ensure confirmability by sharing the final result with two external experts who were familiar with the method of qualitative research and asking them to verify the accuracy of the data encoding process. To achieve dependability, we documented the research process clearly to ensure that it is logical and traceable. In this regard, the whole process of the study was audited by four colleagues. Finally, we have provided an exact explanation of the method to confirm transferability [61].

## Results

**Fig. 2:**
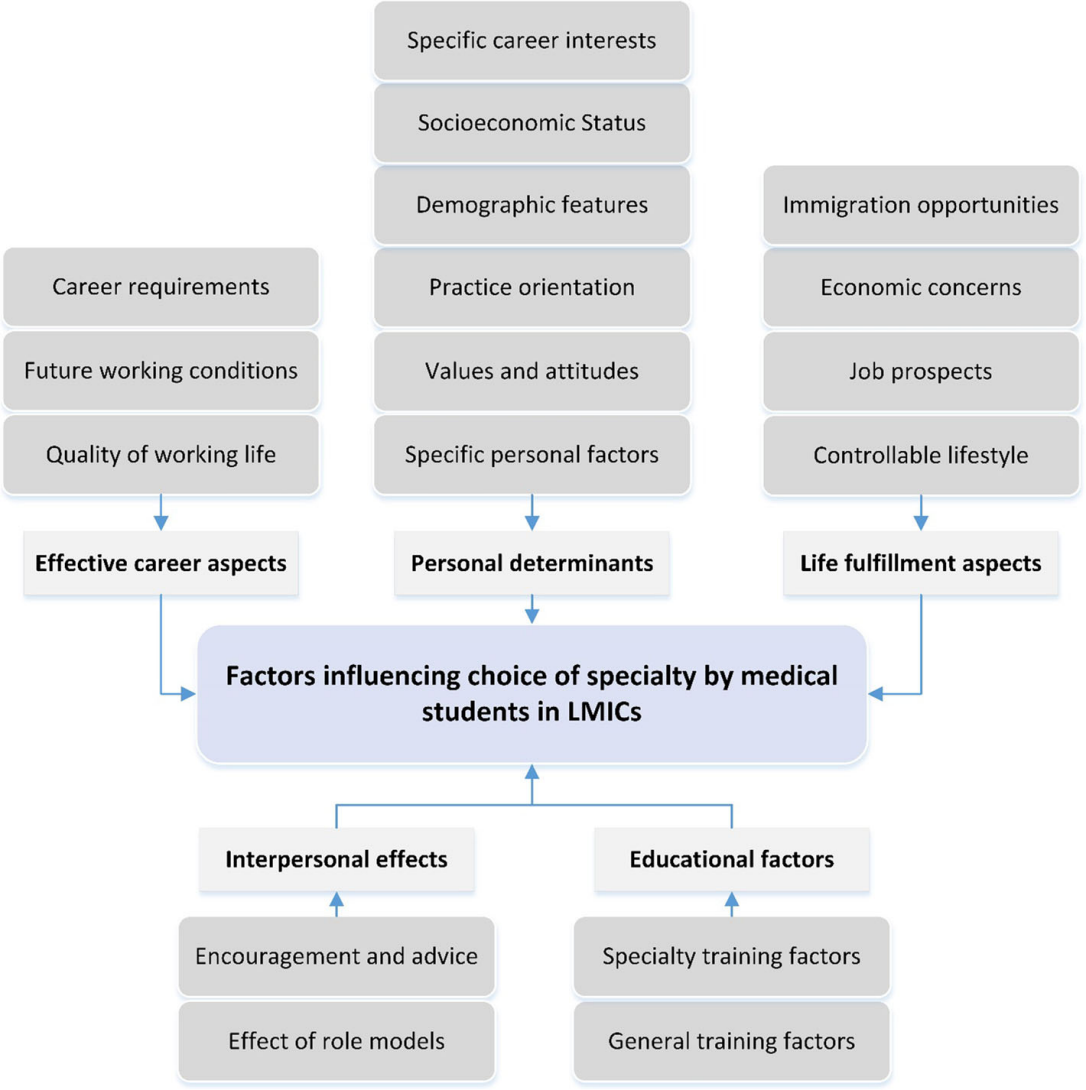
Thematic map of factors influencing the choice of specialty by medical students in LMICs

In this review, we extracted 10,004 articles from the databases, and eventually, 29 studies were included in the final analysis. All studies selected were conducted using a quantitative approach. A large number of articles were from two countries in Asia, including Pakistan (20.6%, N: 6) and India (13.8%, N: 4). Table 2 indicates the characteristics of the selected articles. Thematic analysis resulted in the development of 17 sub-themes and five main themes (Table 3). The main themes that emerged from the analysis are life fulfillment aspects, influential career aspects, personal determinants, educational factors, and interpersonal effects. The results of the thematic analysis are as follows. To avoid duplication of data, we have cited several references for each factor as examples, while a complete list of related references is provided in Table 3. Moreover, Fig. 2 indicates the thematic map of influential factors in the choice of medical specialty by MS.

A- Life fulfillment aspects

**Table 2:** Characteristics of studies included in the synthesis

Characteristics	Frequency (%)
Publication year	
2005–2009	4 (13.8)
2010–2014	13 (44.8)
2015–2020	12 (41.4)
Region	
Africa	7 (24.1)
Asia	17 (58.6)
America	3 (10.3)
Europe	2 (7)
Article type/design	
Quantitative	29 (100)

**Table 3:** Themes and sub-themes on factors influencing the choice of specialty by medical students in LMICs

Themes and Sub-themes	Included Studies	
	Frequency (%)	References
**Life fulfillment aspects**		
Controllable lifestyle	20 (69.0)	[17, 23, 30, 32, 33, 35, 36, 38, 41–50, 52, 56]
Economic concerns	20 (69.0)	[17, 23, 30–33, 35, 36, 39, 41, 42, 44–47, 49–51, 54, 56]
Job prospects	10 (34.5)	[17, 23, 30, 32, 41, 44, 49–52]
Immigration opportunities	1 (3.4)	[51]
**Influential career aspects**		
Quality of working life	18 (62.1)	[17, 23, 31, 33, 35, 36, 38, 39, 41, 42, 44–47, 49–51, 56]
Future working conditions	17 (58.6)	[23, 30–33, 35, 39, 41, 42, 44–47, 49, 51, 52, 56]
Career requirements	15 (51.7)	[17, 23, 30, 33, 35, 36, 41–43, 45–50]
**Personal determinants**		
Specific personal factors	23 (79.3)	[17, 23, 30–32, 34, 35, 37–39, 41–48, 50–52, 54, 56]
Specific career interests	14 (48.3)	[23, 30, 32, 33, 41, 42, 44–47, 49–51, 56]
Values and attitudes	12 (41.4)	[17, 30, 32, 35, 39, 44, 45, 47, 50, 53, 55, 56]
Demographic features	9 (31.03)	[36, 40, 43, 46, 48, 49, 52, 53, 55]
Practice orientation	8 (27.6)	[23, 42, 45–47, 50, 51, 56]
Socioeconomic status	2 (6.9)	[38, 41]
**Educational factors**		
Specialty training factors	13 (44.8)	[17, 23, 32, 39, 41–44, 46, 47, 49, 50, 52]
General training factors	8 (27.6)	[23, 33, 34, 42, 45, 50, 51, 54]
**Interpersonal effects**		
Encouragement and advice	17 (58.6)	[17, 23, 32, 35, 38, 41, 44–47, 49–52, 54, 56]
Effect of role models	15 (51.7)	[17, 23, 31–33, 42, 44–52]

Life fulfillment aspects explain those factors that eventually influence the quality of personal life. These aspects include a controllable lifestyle, economic concerns, job prospects, and immigration opportunities.

*A-1- Controllable lifestyle*: a large number of the studies (69%) reported that controllable lifestyle is an important determinant of specialty selection by MS. Controllable lifestyle is related to working hours [30, 44, 52], having enough personal or leisure time [23, 33, 47], and life stability (including quality of life, and enjoyable life) [38, 48, 49].

*A-2- Economic concerns*: economic and financial concerns are among the highly discussed factors (N: 20, 69%) regarding the choice of specialty. This sub-theme has three categories including future income [31, 41, 56], other financial incentives (such as some financial rewards, and less investment) [41, 44, 54], and status of financial dependency [42].

*A-3- Job prospects*: about 34.5% of the studies (N: 10) pointed out that job prospects determine the choice of specialty by MS. This sub-theme explains the possibility of setting up a personal job or employment opportunities. This factor has two dimensions including job opportunities [17, 23, 51] and job security [32, 41].

*A-4- Immigration opportunities*: only one study [51] reported that international job prospects and immigration opportunities could explain the choice of specialty.

B- Influential career aspects

This theme explains the positive and attractive aspects of a specialty, including future working conditions, quality of working life, and career requirements.

*B-1- Quality of working life*: quality of working life is a concept that refers to the desirability of the job environment. Eighteen studies (62.1%) referred to this factor as a key determinant of specialty choice. Prestige [36, 50], work safety [33, 47], career features (including job satisfaction and climate of working environment) [44, 45], as well as Specialty characteristics (including content of specialty, and extent of interaction with other specialties) [45, 46] are four dimensions of this factor.

*B-2- Future working conditions*: future working conditions are among the most frequent determinants of specialty selection (N: 17, 58.6%). Working conditions reflect the characteristics of a specialty as a career. These conditions include working schedules [46, 51], work-family compatibility [42, 49], autonomy [23, 32], future practice location [35, 39], concern about malpractice consequences [47], and prospects for further development [31, 45].

*B-3- Career requirements*: the effect of future career requirements on the selection of specialty reported by 15 studies (51.7%). This factor explains the particular features of a specialty as a career. These features include diversity of patients [36, 42], extent of interaction with patients [43, 50], existence and amount of on-call or emergency schedule [17, 46], as well as Intellectual challenging field [33, 47].

C- Personal determinants

The “personal determinants” theme includes those features and determinants that are relevant to the students as decision-makers. This theme has six sub-themes, including specific personal factors, specific career interests, demographic features, values and attitudes, practice orientation, and socioeconomic status.

*C-1- Specific personal factors*: this review revealed that the most frequent determinants of specialty selection (N: 23, 79.3%) were personal factors. These factors include personal interest [17, 39, 48], personal competencies [34, 50], personality type [37, 51], personal or relatives experience of a specific disease [38, 42], and personal awareness of the specialty [44, 51].

*C-2- Specific career interests*: fourteen studies (48.3%) indicated that interest in specific careers explains the choice of specialty. This sub-theme has three categories, including the prospect of procedural work [33, 51], teaching career prospects [41, 49], and interest in research [23, 46].

*C-3- Demographic features:* some studies (N: 9, 31.03%) emphasized the impact of demographic features. These variables include gender [53, 55], hometown or current living location [40, 48].

*C-4- Values and attitudes:* values ​​and attitudes that are mainly related to the cultural and philosophical context of individuals could largely determine the choice of specialty (N: 12, 41.4%). These include empathy [53, 55], altruism [35, 39], and social responsibility [32, 50].

*C-5- Practice orientation:* practice orientation implies the interest of individuals in specific medical practice. Findings of eight studies (27.6%) indicated two main categories of this factor, including hospital or treatment orientation [51] and community or prevention orientation [46, 50].

*C-6- Socioeconomic status:* only two studies highlighted the role of socioeconomic status of MS and their families in the choice of specialty. This factor is related to the income level of students’ family and occupation status of their family members [44, 51].

D- Educational factors

This theme illustrates the influence of some factors related to the general and specialty training courses.

*D-1- General training factors*: factors that are related to the general training course could determine the choice of specialty by MS (N: 8, 27.6%). These factors include medical school type (public or private) [34], influence of educational environment such as policies of medical collages [47], as well as educational and clinical experiences [42, 54].

*D-2- Specialty training factors*: thirteen studies (44.8%) reported that factors related to specialty training courses are also influential in specialty selection. This sub-theme comprises five categories, including length of the training period [32, 43], difficulty of the training period [32, 39], competitiveness of the specialty [41, 49], availability of specialty courses [44, 52], and lifestyle of residency period [42, 47, 49].

E- Interpersonal effects

Interpersonal effects reflect the influence of different individuals or groups on decisions regarding specialty selection. These effects are related to role models and encouragement or advice from others.

*E-1- Effect of role models*: some studies (N: 15, 51.7%) emphasized the effect of role models on the selection of specialty by MS. Family members or friends who have educated in a particular field [44, 48] as well as pleasant experience with teachers and other physicians [45, 47] could be influential in this regard.

*E-2- Encouragement and advice*: encouragement and advice from others are among the interpersonal factors that explain the choice of specialty. Some studies (N: 17, 58.6%) showed that advice from family members [23, 50], friends’ advice [32, 56], advice from senior students and teachers [46, 54], influence of mentors [32], as well as encouragement and expectations of the society [50], are the main aspects.

## Discussion

Choice of medical specialty is a complex process and has many factors involved. Factors that affect medical specialty choice have been explored in the literature either in relation to specific specialties or by focusing on a limited number of determinants. Due to the importance of an in-depth understanding of the factors affecting specialty choice for effective physician workforce policy, in this review, we aimed to provide a comprehensive map of the evidence without a focus on a particular specialty. Moreover, we studied these factors to determine the research gaps and the areas for further investigations.

The synthesis of data led to the development of five main themes regarding the factors influencing specialty choice by MS. These include life fulfillment aspects, influential career aspects, personal determinants, educational factors, and interpersonal effects. These findings are in line with the results of other review studies that have been conducted on particular specialty fields [8, 62–65].

The findings revealed that the most frequent sub-themes regarding the choice of specialty were specific personal factors, controllable lifestyle, quality of working life, and future working conditions. The thematic map that was developed in this study could be used by policymakers in LMICs in reform programs aimed at achieving more balanced distribution of physicians, increasing the availability of services at all levels of health care, and ensuring a sustainable supply of physician workforce based on community health needs. However, due to contextual differences, the weight and interrelationships of these factors should be assessed in each country separately before formulating any policy.

Based on the findings of these reviews and the studies analyzed in this review, it could be concluded that many factors identified in this study should be explained in terms of two underlying features, including gender and medical specialty categories (primary care versus non primary care specialties). Some factors are more influential in the choice of specialty by a particular gender group. Also, the importance of factors varies between the choice of primary care specialties (such as general practice and family physician) and other specialty fields.

### Life fulfillment aspects

This study showed that controllable lifestyle is an important determinant of specialty choice that is consistent with the findings of two other reviews [8, 66]. This factor is mainly explained by the compatibility of working hours with leisure time and personal life. Some studies indicated that the weight of this aspect is higher in the selection of primary care specialties and among females [65, 66]. Although, some studies reported equal importance of the factor for both genders [67, 68]. Increasing the flexibility of working hours in other specialties [66] as well as the higher impact of other factors may be the reasons for this discrepancy.

This review indicated that economic concerns have a great effect on the choice of specialty. This finding is in line with the results of other relevant reviews [8, 62, 69]. It is argued that financial factors such as future income are more important in the selection of non-primary care specialties such as surgery [8, 64]. Some evidence suggests that income may not be considered as a high priority goal by females when choosing a specialty [62, 70]; However the effect of other factors may modify this condition [71]. Financial dependency and level of debt are two examples of these factors [8, 20, 72]. Financial factors can be considered as an important determinant of the shortage of physicians in some specialties, which poses serious risks to the provision of services in many LMICs. Moreover, it should be noted that the effect of preference for high-earning specialties in LMICs and particularly developing countries could be assessed in relation to some factors such as immigration tendencies and the brain drain phenomenon.

The findings revealed that an acceptable job prospect is an attractive feature of a specialty. A considerable number of studies have addressed this issue. However, other review studies in this area have not reported this factor. It seems that in some countries, availability of job and permanent job security are located in the focus of attention due to the saturation of job market in some specialties [73], employment obstacles [44], and difficulties in launching a new private job [7].

This review showed that only one study had examined the impact of immigration tendencies on the choice of specialty [51]. Based on our searches, no other review study has reported this issue. The immigration of physicians to more prosperous countries is a critical challenge for many developing countries. This type of outflow could aggravate physicians’ imbalances in LMICs [74]. Immigration of physician workforce from LMICs could have profound impact on economic growth, human development [75], and the potentiality of health systems in addressing the needs of community. Therefore, the need for comprehensive investigations in this regard is indubitable.

### Influential career aspects

This study revealed that future working conditions have a great impact on specialty selection. Consistent with the results of this study, most of these conditions have been elucidated by other review studies. These reviews showed that working schedule [8, 65, 66] and work-family compatibility [65, 66] are more important for women and also weigh more in choosing primary care specialties. On the contrary, prospects for further development [66] and the possibility to work in urban areas [63] are more compatible with non-primary care specialties. It seems that autonomy and concern about malpractice consequences, as two other working conditions, have the same influences in choosing various specialties and in different gender groups.

Quality of working life is one of the leading determinants of specialty choice. Prestige is one aspect of this factor that other review studies have also confirmed it. According to those studies, the role of prestige is more prominent in the choice of non-primary care specialties [8, 64]. It is also not recognized as a top priority among females [70]. Occupational hazards, job burnout, and job satisfaction are among the specific career features that have not been directly addressed in other review studies. Although, some of them have implicitly pointed to the higher impact of these factors on the choice of specialty by females and in the selection of primary care careers [8, 66].

Our study clarified the impact of specific career requirements on specialty selection. Similar to our study, one review study showed that the extent of patient-physician interaction could influence the choice of specialty, particularly in primary health care careers [8]. Consistent with the findings of our study, some review articles reported that the diversity of patients and activities is a predictive factor in the choice of specialty. It is suggested that those who are more interested in the diverseness of activities are more likely to pursue a primary care specialty [8, 62]. Another requirement of a medical specialty is emergency or on-call schedules. This aspect was not reported in other review articles. However, it seems that there is a close relationship between the on-call schedule and some other factors such as flexibility of working schedule, controllable lifestyle, and job burnout. Therefore, it could be concluded that this factor is more influential in the selection of specialty by females. Intellectual challenge is another requirement of a specialty that is suggested to be more important in the selection of academic careers [76] and surgical specialties [65].

### Personal determinants

We found that personal interest, personal competencies, personal experience of a disease, personality traits, as well as personal awareness of the specialty are the most frequent determinants of specialty selection. Although some reviews suggested that certain personality traits may predispose individuals to the selection specific specialties [8, 65, 70], others have argued that there is insufficient evidence to substantiate this claim [63, 66, 77]. A cross-sectional study reported that personality traits should be taken into account when considering the possibility of interaction with other factors. This may explain the difference in the findings [68]. The role of other specific personal factors was not stipulated in the reviews we checked. However, it can be assumed that many other determinants such as special career interests [62, 76, 77], particular practice orientation [63], as well as some values and attitudes [8, 66, 77] that are mentioned by these reviews are mainly under the influence of these underlying personal factors. A review study reported that practice orientation toward prevention and community health is influential in the choice of a career in the primary care fields [63]. Also, some reviews specified that interest in research and academic teaching [8, 63, 76, 78] as well as preference for new technologies and procedural works [62, 65] are more compatible with non-primary care careers.

In line with the results of our study, other reviews emphasized the effect of demographic characteristics on the selection of specialty. It is suggested that being female [8, 62, 64–66, 70], the experience of living in rural areas [8, 63], being married, and being older [8, 62] are more associated with the selection of primary care specialties. As we discussed above, these findings could be explained by some factors such as controllable lifestyle, work-family compatibility, and future working conditions. Some reviews, consistent with our study, pointed to the effect of specific values and attitudes such as altruism, empathy, and social responsibility in the choice of medical specialties. These studies point to the higher weight of these values in choosing primary care specialties [8, 70, 77]. Furthermore, some of the reviews have implicitly pointed to the more prominent role of these factors among females [8, 70]. This review, similar to another relevant review [5], showed that individuals’ socioeconomic status influences their choice of specialty. The mentioned review has reported that individuals from families with higher socioeconomic status are more interested in non-primary care specialties. This may be related to individuals’ lifestyle, living location, and family members’ education level.

### Educational factors

Similar to our study, some reviews have reported the influence of factors related to general education course including positive educational and clinical experiences [8, 65, 77, 79], the influence of university environment and policies [8, 63, 78], educational curriculum effects [8], type of medical schools [8, 63], as well as place of education [8, 77]. Some of these studies suggested that private medical schools [8, 63], training in big cities [8, 77], treatment-based curriculums [8], as well as university policies toward more clinical specialties [8, 63, 78] are predictors of the selection of careers in non-primary care fields. It seems that private schools encourage individuals to choose higher-earning specialties because of imposing a high financial burden on them [8]. Also, it could be suggested that education in big cities shapes the expectation of people to choose specialties that are more compatible with the urban areas.

We found that educational aspects of specialty courses influence the choice of specialty. Some review studies, consistent with our findings, have indicated that the length and difficulty of the specialty [8, 64, 79] as well as residency lifestyle [64] are among these factors. These studies showed that the two mentioned factors were barriers for individuals to choosing non-primary care specialties. However, specialty competitiveness and availability of training courses have not been reported by other reviews.

### Interpersonal effects

In line with the results of our study, some reviews showed that role models [64, 65, 78], as well as advice and encouragement of others [66, 77], affect the choice of specialty. Based on the results of different reviews, it could be concluded that the influence of role models and advice on the selection of various specialties are the same in both genders.

### Research gaps

In this review study, we identified some research gaps. Firstly, all of the included studies have been cross-sectional. Quantitative studies, compared to qualitative studies, have a limited potentiality to provide a more in-depth insight into the factors associated with an issue [80]. Therefore, to provide more precise evidence, further qualitative and mixed-method studies are of great importance. Secondly, despite the fact that a large number of studies investigated factors affecting the choice of specialty, no studies have investigated the weight of these factors. Accordingly, in order to provide appropriate evidence for policymaking, it is necessary to explore the weight, rank, and interrelationships between these factors in different socioeconomic contexts. As the third gap, we found that immigration to developed countries, despite its importance, has not received much attention from researchers in studies aimed to determine factors influencing the choice of specialty [51]. Thus, importance of immigration tendencies in the selection specialty and factors associated with this phenomenon are areas for further investigations in LMICs. The final gap is that despite some efforts, little attention has been paid to the influence of cultural variables and broader concepts such as ethical values and medical philosophy aspects.

### Strength and limitations of the study

As a strength, in this review, we tried to draw a comprehensive map of the evidence on the factors influencing specialty selection by MS. The results of the study can be used as evidence for health human resource planning. Moreover, although assessment of the quality of articles is not considered as a part of the scoping review methodology, we conducted a quality appraisal of all selected studies using standard checklists in order to provide more precise evidence. One limitation of this study is that due to the language limitations, we only selected studies with a full-text in English. This limitation causes loss of evidence published in other languages. Also, although we tried to do a comprehensive search, it is inevitable that some articles may be missed unintentionally. Another limitation of this review is that we only focused on studies conducted among MS with clinical experiences, so evidence from studies carried out among MS in other educational grades are not included. Finally, in this review we used thematic content analysis, the various range of data collection and analysis methods used in the selected studies makes it difficult to synthesize the results.

## Conclusion

Specialty selection is a complex phenomenon with many factors involved. This review study provided evidence on the factors influencing the choice of medical specialties by MS in LMICs that could be useful for policymaking. Accordingly, life fulfillment aspects, influential career aspects, personal determinants, educational determinants, and interpersonal effects were developed as the main themes. Furthermore, the most frequent sub-themes were controllable lifestyle, economic concerns, specific personal aspects, demographic features, quality of working life, and future working conditions. This review revealed that the effect of some factors on the selection of specialty remains areas for further investigations. These factors include ethical values, various aspects of medical philosophy, and interest in immigration to developed countries. Based on the results of this review, it could be suggested that the socioeconomic contexts explain the importance and weight of factors associated with specialty selection. Therefore, in order to support evidence-based policymaking, countries must determine the weight, ranking, and the interrelationships between these factors based on their specific circumstances. Accordingly, mixed-method studies, that are useful tools for generating the evidence, should be of more interest to researchers.

## Supplementary Information

**Additional file 1.**

**Additional file 2.**

## Data Availability

The data charting form and the thematic analysis tables are provided in the form of additional files.

## Abbreviations

HICs: High-income countries
LMICs: Low-and, middle-income countries
MS: Medical Students
PCC: Population, Concept, and Context
STROBE: Strengthening the Reporting of Observational Studies in Epidemiology
CASP: Critical Appraisal Skills Programme
