# Supplementary material for: A thematic network for factors affecting the choice of specialty education by medical students: a scoping study in low-and middle-income countries
Source: BMC Med Educ. 2021 Feb 10;21:99. doi: 10.1186/s12909-021-02539-5 (PMC7877062; doi:10.1186/s12909-021-02539-5)
Supplement: Supplementary file 1 — Additional file 1. [file 12909_2021_2539_MOESM1_ESM.docx]

Data from articles published on factors associated with specialty selection among medical students in LMICs

| **No** | **Author (?)**  **Country** | **Study Title** | **Journal**  **Year** | **Study Design**  **Sample Size** | **Key Determinants of Specialty Selection** |
| --- | --- | --- | --- | --- | --- |
| 1 | Sabiha et al.  Pakistan | A Cross Sectional Study on the Choices of Female Medical Students in Selection of Their Future Specialties | Journal of Postgraduate Medical Institute  2016 | Quantitative  300 | Personal Interest  Inspiration from a Role Model  Specialty is Considered Socially Appropriate for The Female  Charm of The Field  Easy Life Style in Future/Convenience of Working Hours  Job Prospects in Future  Prospect of Private Practice In Future  Pressure from Family/Friends  Availability of Training Slots |
| 2 | Ahmed et al.  Bangladesh | Career choices among medical students in Bangladesh | Advances in Medical Education and Practice  2011 | Quantitative  132 | Having time for spouse and family  Having option to practice in rural community  Having academic/teaching prospect  Wide job opportunity  Availability of postgraduate training  Having time for other personal interests  Having fixed hours of work  Having option to help people more  Having a direct dealing with patients  Income will allow an enjoyable lifestyle  Personal interest |
| 3 | Hayes et al.  Nepal | Career choices and what influences Nepali medical students and young doctors: a cross-sectional study | Human Resources for Health  2013 | Quantitative  1112 | Serve sick  Personal interest  Social prestige  Employment prospects  Best available course  Serve rural areas  Self/government employment  Scientific/research interest  Financial remuneration  Financial remuneration –during PG study  Financial remuneration – after PG study  Personal or family Illness  Books/films  Family pressure  Same doctor in family  Work satisfaction  Opportunity for procedural work  Work hours  Pursuit of non-medical interests |
| 4 | Dossajee et al.  Kenya | Career preferences of final year medical students at a medical school in Kenya–A cross sectional study | BMC Medical Education  2016 | Quantitative  156 | Hometown (urban origin, rural origin) |
| 5 | Huda et al.  Pakistan | Career Preference of Final Year Medical Students of Ziauddin Medical University | Education for Health  2006 | Quantitative  232 | Personal interest  Role models in medical college  Clinical rotation  Intellectual challenge  Professional independence  Close interaction with patients  Content of specialty  Desired practice setting  Fixed working hours  Specialty prestige  Financially rewarding  Parental preference  Commitment to family /community  Burden of disease  Few specialists in the country  Working with new technology  Opportunities for contribution to society  Opportunities for research  Policies/mission of medical college |
| 6 | Azu et al.  South Africa | Choice of specialty amongst first-year medical students in the Nelson R. Mandela School of Medicine, University of KwaZulu-Natal | African Journal of Primary Health & Family Medicine  2013 | Quantitative  167 | Benefits to patients  Personal Interest  Challenge  Financial  Lifestyle  Working Lifestyle  Working hours  Like urban area  Status/Prestige  Like Rural area  Parental Influence |
| 7 | Tariq et al.  Pakistan | Differences in Empathy Levels of Medical Students Based on Gender, Year of Medical School and Career Choice | Journal of the College of Physicians and Surgeons Pakistan  2018 | Quantitative  337 | Students with interest in medicine and allied showed higher empathy scores compared to surgical or technical specialties. |
| 8 | Santos et al.  Brazil | Empathy differences by gender and specialty preference in medical students: a study in Brazil | International Journal of Medical Education  2016 | Quantitative  320 | Our study has found consistently high scores of empathy among medical students enrolled in all levels of training at the Federal University of Santa Catarina, and higher empathy among women and students who intend to pursue a people-oriented specialty. |
| 9 | Dikici et al.  Turkey | Factors Affecting Choice of Specialty Among First-year Medical Students of Four Universities in Different Regions of Turkey | Croatian Medical Journal  2008 | Quantitative  717 | Money and prestige  Personal development  Benefits for the patient  Wish to work in urban area  Easiness  Personal interest |
| 10 | Khader et al.  Jordan | Factors affecting medical students in formulating their specialty preferences in Jordan | BMC Medical Education  2008 | Quantitative  440 | Gender  Hours of practice  On-call schedule  Flexibility of specialty  Interaction with physicians  Specialty reputation  Duration of residency program  Work pressure  Interest in research  Interest in long term relations with patients  Physician-patient interaction  Diversity of patients  Anticipated income  Focus on community health  Focus on urgent care Curriculum  Intellectual content of the specialty  Individual's competencies  Emulate a physician  Advice from faculty  Advice from friends  Advice from parents  Advice from practicing physicians |
| 11 | Alawad et al.  Sudan | Factors considered by undergraduate medical students when selecting specialty of their future careers | Pan African Medical Journal  2015 | Quantitative  647 | Personal interest  Helpful to the community  Job opportunities  Financial reasons  Advice from others  Prestigious  Shorter work hour  Avoid on-calls  Shorter length of training |
| 12 | Kumar et al.  Multi-country  (China, Sri Lanka, Nepal, India and Malaysia) | Factors Influencing Medical Students’ Choice of Future Specialization in Medical Sciences: A Cross-Sectional Questionnaire Survey from Medical Schools in China, Malaysia and Regions of South Asian Association for Regional Cooperation | North American Journal of Medical Sciences  2014 | Quantitative  1470 | Gender  Parents education in the field  Origin (urban, rural)  Intellectual content  Interesting  Quality of life  Early experience |
| 13 | Bhat et al.  India | Factors Influencing the Career Choices of Medical Graduates | Journal of Clinical and Diagnostic Research  2012 | Quantitative  250 | Gender  Income  Prestige  Hazards  Fixed working hours  Stress  Scope of practice |
| 14 | Anand et al.  India | Factors influencing the career preferences of medical students and interns: a cross-sectional, questionnaire-based survey from India | Journal of Educational Evaluation for Health Professions  2019 | Quantitative  368 | **Personal growth:**  Flexible working hours  Less stressful working conditions  Less duration of work hours  Comfortable lifestyle  Sufficient time for hobbies and personal interests  Family responsibilities  **Professional growth:**  Opportunities for higher studies or further specialization  Opportunity to do research  Perceived status of the field  Opportunity to settle down in urban areas  Professionally challenging  Financial prospects  **Personal satisfaction:**  Opportunity to be involved in patient care Preference to work in rural areas  Opportunity to teach  Influences from past experiences  Influenced by role models |
| 15 | Khater-Menassa et al.  Lebanon | Factors influencing the choice of specialty among medical students in Lebanon | The Lebanese Medical Journal  2005 | Quantitative  127 | **1. MATCH OF PERSONAL INTEREST/SKILLS**  • Consistent with personality  • Diversity in diagnosis and therapy  **2. HELPING/SOCIAL RESPONSIBILITY**  • Interest in helping people  **3. INTELLECTUAL OPPORTUNITIES**  • Intellectual content of the specialty  • Challenging diagnostic problems  • Opportunity for research  • Working with new technology  • Keeping options open for subspecialty  **4. PATIENT CONTACT**  • Type of patients  • Desire to do ambulatory care  • Emphasis on patient education and prevention  **5. ENCOURAGEMENT/ROLE MODELS**  • Role model of a physician in the specialty  • Opinion of other students/residents  **6. L IFESTYLE ATTRIBUTES**  • Sufficient time for family/personal activities  • Predictable working hours  • Lack of stress in the field  **7. CLERKSHIP/COURSES**  • Availability of residency program locally  • Availability of residency program internationally  • Ease of enrollment in residency program locally  • Ease of enrollment in residency program internationally  **8. LEADERSHIP AND PRESTIGE**  • Opportunity to lead  • Prestige of chosen specialty  **9. RESIDENCY ISSUES**  • Not enough field specialists  • Length and lifestyle of residency  **10. ECONOMIC ISSUES**  • Income prospect  • Malpractice insurance costs |
| 16 | Ligia et al.  Brazil | Medical Specialty Choice and Related Factors of Brazilian Medical Students and Recent Doctors | PloS one  2015 | Quantitative  1225 | Lifestyle  Perceived ability  Way of wok  Autonomy  Variety of medical problems  Internship (curricular)  Role models  Financial reason  Academic experience  Personal time  Social commitment  Prestige of specialty  Residency time  Research opportunity  Family influence |
| 17 | Seyoum et al.  Ethiopia | Medical students' choice of specialty and factors determining their choice: a cross-sectional survey at the Addis Ababa University, School of Medicine, Ethiopia | Ethiopian Medical Journal  2014 | Quantitative  161 | Inspiration during their clinical practice  Financial reward  Dedication to the field  Possession of competency  Influence of teacher |
| 18 | Rehman et al.  Pakistan | Pakistani medical students’ specialty preference and the influencing factors | Journal of Pakistan Medical Association  2011 | Quantitative  771 | Financially highly paid specialty  Field requiring surgical skills/work  Willingness to work in hospital  To work as a General Practitioner/Primary Care Physician  Suited to an individual personality  Awareness of time and work before choosing  Prestige labeled to a specialty  Academic results in the same specialty  Peer pressure ( friend's choice)  Jobs availability  Parent's influence  Personal health  Impressed by a role-model  Flexibility (can change their specialty choice)  Scope of a specialty internationally |
| 19 | Dhodi et al.  India | Personal and family factors influencing specialty preference of medical students | Indian Journal of Health and Wellbeing  2017 | Quantitative 827 | Self interest  Self-satisfaction  Name and fame  Lifestyle  Health history  **Family factors:**  Parental wishes  Family income  Care of the family  Health history of family member  Family occupation |
| 20 | Budakoglu et al.  Turkey | Personality and Learning Styles of Final-Year Medical Students and the Impact of these Variables on Medical Specialty Choices | Gazi Medical Journal  2014 | Quantitative  170 | Last-year medical students are characterized by a ISTJ personality type in most of the medical specialty preferences |
| 21 | Hamid et al.  Pakistan | Specialty preference with respect to gender among medical students of Pakistan | Journal of the Pakistan Medical Association  2019 | Quantitative  314 | Gender  Interest in content  Good Salary  Direct Patient Interaction  Convenient on-call schedule  Minimum working hours  Shorter duration of residency programs |
| 22 | Gutiérrez-Cirlos et al.  Mexico | Specialty choice determinants among Mexican medical students: a cross-sectional study | BMC Medical Education  2019 | Quantitative  697 | **Personal values during undergraduate training:**  Interest in the specialty patient type  Variety of medical problems in the specialty  Work to do during the specialty  Specialty social engagement  Possibility of studying a subspecialty  Opportunities to perform research  **Career needs to be satisfied:**  Specialty duration  The expectation of free time  Possibility of raising a family  Potential autonomy after graduation  Financial reasons  Family support during the specialty  **Perception of the specialty characteristics:**  Role models  Pleasant academic experience in the specialty  Medical internship with pleasant  experiences in the specialty  Specialty prestige  To have skills related to the specialty |
| 23 | Aslam et al.  Pakistan | Specialty choices of medical students and house officers in Karachi, Pakistan | Eastern Mediterranean Health Journal  2011 | Quantitative  909 | Type of collage (Public, Private) |
| 24 | Onyemaechi et al.  Nigeria | Specialty Choices: Patterns and Determinants among Medical Undergraduates in Enugu Southeast Nigeria | Nigerian Journal of Clinical Practice  2017 | Quantitative  152 | Personal interest  Prestige of specialty  Anticipated income  Good clerkship experience  Personal abilities/competence  Effect of role model  Parental advice  Teachers advice  Friends advice  Family/societal expectations  Career prospects  Shortage of specialist  Less competitive field  Low work hours  Short period of training  Research opportunity  Low work‑related risks  Diversity of patients  Focus on community health  Physician patient relationship |
| 25 | Gour et al.  India | Specialty Preference Among Medical Students and Factors Affecting It | Online Journal of Health and Allied Sciences  2013 | Quantitative  190 | More income  Job security  Early settlement  Interest  Less investment  Specialty reputation  Less competition  Less hours of practice  For teaching as hobby  Afraid of operative procedures/blood  More scope  Preference of terminal branch  Relationship with patients  Intellectual content of specialty  No on call/emergency schedule  Influenced by friends  Advice from teachers  Family pressure  Family background |
| 26 | Maseghe Mwachaka et al.  Kenya | Specialty preferences among medical students in a Kenyan university | Pan African Medical Journal  2010 | Quantitative  385 | Encouragement by staff  Role model in the specialty  Job and financial rewards  Prestige of the specialty  Lifestyle of practice  Ease of raising a family  Intellectual challenge  Length of residency training  Ease of entry into residency  Lifestyle during residency  Further training after residency  Gender distribution in specialty  Academic or research opportunities |
| 27 | Ambrose et al.  Botswana | Specialty preferences among medical students in Botswana | BMC Research Notes  2017 | Quantitative  116 | Personal interest and aptitude  Financial reward  Role model and mentor effect  Shortage of specialists in that field  Job security  Prospect of self-employment  Potential to do research  Influence of parents, guardians, friends  Time for other things in life  Short learning period  Specialty not currently offered  Convenience  altruism |
| 28 | Al-Mendalawi et al.  Iraq | Specialty preferences of Iraqi medical students | The Clinical Teacher  2010 | Quantitative  93 | Personal interest  Anticipated higher income  Prestige  Anticipated future mastering skills and development  Charismatic role models  Family or spousal influence |
| 29 | Vahid Dastjerdi et al.  Iran | Study Motives and Career Choices of Iranian Medical and Dental Students | Acta Medica Iranica  2012 | Quantitative  350 | **Social and professional status:**  Income  Work independence  Social status  Social and matrimonial considerations  **Health care and people:**  Helping people to improve their health  Playing a role in community health promotion  Interest in research  **Others' recommendation:**  Parents' recommendation  Friends' advice  Career advisors  **personal interest and nature of occupation:**  Personal interest  Combination of practical and theoretical skills  **Occupational experience:**  Personal experience  Influence of parents  **Personal life:**  Devoting more time to self and family  Occupational stress |
