# Supplementary material for: A thematic network for factors affecting the choice of specialty education by medical students: a scoping study in low-and middle-income countries
Source: BMC Med Educ. 2021 Feb 10;21:99. doi: 10.1186/s12909-021-02539-5 (PMC7877062; doi:10.1186/s12909-021-02539-5)
Supplement: Supplementary file 2 — Additional file 2. [file 12909_2021_2539_MOESM2_ESM.docx]

**Thematic Analysis (Initial codes & categories)**

| **No** | **Categories** | **Initial Codes (Article number in data charting form)** |
| --- | --- | --- |
| 1 | Personal Competencies | Individual's competencies (10)  Perceived ability (16)  Possession of competency (17)  To have skills related to the specialty (23)  Personal abilities/competence (24)  Personal aptitude (27) |
| 2 | Academic career prospect | Having academic/teaching prospect (2)  Opportunity to teach (14)  For teaching as hobby (25)  Academic or research opportunities (26) |
| 3 | Job opportunities | Job prospects in future (1)  Wide job opportunity (2)  Employment prospects (3)  Job opportunities (11)  Way of wok (16)  Jobs availability (18)  Career prospects (24)  Job rewarding (26) |
| 4 | International job opportunities | Scope of a specialty internationally (18) |
| 5 | Job security | Job security (25)  Job security (27) |
| 6 | Family advice | Pressure from family (1)  Family pressure (3)  Parental preference (5)  Parental Influence (6)  Advice from parents (10)  Family influence (16)  Parent's influence (18)  Parental wishes (19)  Family expectations and parental advice (24)  Family pressure (25)  Influence of parents (27)  Family or spousal influence (28)  Parents' recommendation and Influence of parents (29) |
| 7 | Teachers advice | Advice from faculty (10)  Influence of teacher (17)  Teachers advice (24)  Advice from teachers (25) |
| 8 | Senior students’/doctors’ advice | Advice from practicing physicians (10)  Opinion of other students/residents (15) |
| 9 | Friends’ advice | Pressure from friends (1)  Advice from friends (11)  Peer pressure ( friend's choice) (18)  Friends advice (24)  Influenced by friends (25)  Influence of friends(27)  Friends' advice (29) |
| 10 | Advice from others | Advice from others (11)  Encouragement by staff (26)  Influence of guardians (27)  Career advisors (29) |
| 11 | Society expectations | Societal expectations (24) |
| 12 | Teacher/other physicians role models | Inspiration from a role model (1)  Role models in medical college (5)  Emulate a physician (10)  I’m trying to become like a doctor known to me (11)  Influenced by role models (14)  Role model of a physician in the specialty (15)  Role models (16)  Impressed by a role-model (18)  Role models (22)  Effect of role model (24)  Role model in the specialty (26)  Role model and mentor effect (27)  Charismatic role models (28) |
| 13 | Family/ friend role models | Same doctor in family (3)  Parents education in the field (12) |
| 14 | Educational and clinical experience | Clinical rotation (5)  Influences from past experiences (14)  Internship (curricular)/ Academic experience (16)  Inspiration during their clinical practice (17)  Academic results in the same specialty (18)  Pleasant academic experience in the specialty/Medical internship with pleasant (22)  Good clerkship experience (24) |
| 15 | Educational environment influence | Policies/mission of medical college (5) |
| 16 | Prospect of Procedural work | Opportunity for procedural work (3)  Opportunity to be involved in patient care (14)  Field requiring surgical skills/work (18)  Afraid of operative procedures/blood (25)  Combination of practical and theoretical skills (29) |
| 17 | Work safety | Work pressure (10)  Hazards and stress (13)  Less stressful working conditions (14)  Lack of stress in the field (15)  Low work‑related risks (24)  Occupational stress (29) |
| 18 | Length of Training period | Length of training to consultancy (10)  Shorter length of training (11)  Length (15)  Residency time (16)  Shorter duration of residency programs (21)  Specialty duration (22)  Short period of training (24)  Length of residency training (26)  Short learning period (27) |
| 19 | Difficulty of the training period | Easiness (9)  Convenience (27) |
| 20 | lifestyle of residency | lifestyle of residency (15)  Work to do during the specialty (22)  Lifestyle during residency (26) |
| 21 | Income | Income will allow an enjoyable lifestyle (2)  Money (9)  Anticipated income (10)  Income (13)  Income prospect (15)  Financially highly paid specialty (18)  Good Salary (21)  Anticipated income (24)  More income (25)  Anticipated higher income (28)  Income (29) |
| 22 | Financial incentives | Financial remuneration/Financial remuneration –during PG study/Financial remuneration after PG study (3)  Financially rewarding (5)  Financial issues (6)  Financial reasons (11)  Financial prospects (14)  Financial reason (16)  Financial reward (17)  Financial reasons (22)  Early settlement / Less investment (25)  Financial rewards (26)  Financial reward (27) |
| 23 | Possibility of private working | Prospect of private practice In future (1)  Self/government employment (3)  Professional independence (5)  Prospect of self-employment (27)  Work independence (29) |
| 24 | Autonomy | Autonomy (16)  Potential autonomy after graduation (22) |
| 25 | Overall future lifestyle | Easy life style in future (1)  Enjoyable lifestyle (2)  Lifestyle and working lifestyle (6)  Comfortable lifestyle (14)  Lifestyle (16)  Lifestyle (19)  Lifestyle of practice (26) |
| 26 | Prestige | Social prestige (3)  Specialty prestige (5)  Status/Prestige(6)  Prestige (9)  Specialty reputation (10)  Prestigious (11)  Prestige (13)  Perceived status of the field(14)  Prestige of chosen specialty / Opportunity to lead (15)  Prestige of specialty (16)  Prestige labeled to a specialty (18)  Name and fame (19)  Specialty prestige (22)  Prestige of specialty (24)  Specialty reputation (25)  Prestige of the specialty (26)  Prestige (28)  Social status (29) |
| 27 | Diversity of patients | Diversity of patients (10)  Scope of practice (13)  Type of patients/ Diversity in diagnosis and therapy(15)  Variety of medical problems (16)  Variety of medical problems in the specialty (22)  Diversity of patients (24)  More scope (25) |
| 28 | Gender | Specialty is considered socially appropriate for the female (1)  Gender (7), (8), (10), (12), (13), (21)  Gender distribution in specialty (26) |
| 29 | Working schedules | Flexibility of specialty (10)  Flexibility (18) |
| 30 | Hospital orientation | Willingness to work in hospital (18) |
| 31 | Community orientation | Focus on community health (5)  Focus on community health (10)  Emphasis on patient education and prevention (15)  Social commitment (16)  To work as a general practitioner/Primary care physician (18)  Specialty social engagement (22)  Focus on community health (24)  Playing a role in community health promotion (29) |
| 32 | Working hours | Convenience of working hours (1)  Having fixed hours of work (2)  Work hours (3)  Fixed working hours (5)  Working hours (6)  Hours of practice (10)  Shorter work hour (11)  Fixed working hours (13)  Flexible working hours/ Less duration of work hours (14)  Predictable working hours (15)  Minimum working hours (21)  Low work hours (24)  Less hours of practice (25) |
| 33 | Interest in research | Scientific/research interest (3)  Opportunities for research (5)  Interest in research (10)  Opportunity to do research (14)  Opportunity for research (15)  Research opportunity (16)  Opportunities to perform research (22)  Research opportunity (24)  Research opportunities (26)  Potential to do research (27)  Interest in research (29) |
| 34 | Intellectual challenging area | Intellectual challenge/Working with new technology (5)  Challenge (6)  Intellectual content of the specialty (10)  Intellectual content (12)  Professionally challenging (14)  Intellectual content of the specialty/Challenging diagnostic problems/ Working with new technology (15)  Intellectual content of specialty (25)  Intellectual challenge (26) |
| 35 | Personality type | Consistent with personality (15)  Suited to an individual personality (18)  Personality (20) |
| 36 | Extent of interaction with patients | Having a direct dealing with patients (2)  Having a direct dealing with patients (5)  Interest in long term relations with patients/ Physician-patient interaction (10)  Direct Patient Interaction (21)  Physician patient relationship (24)  Physician patient relationship (25) |
| 37 | Empathy | Empathy(7)  Empathy (8) |
| 38 | Living location (Hometown) | Hometown (urban origin, rural origin) (4)  Origin (urban, rural) (12) |
| 39 | Future practice location | Having option to practice in rural community (2)  Serve rural areas (3)  Like Rural area / Like urban area (6)  Wish to work in urban area (9)  Opportunity to settle down in urban areas/ Preference to work in rural areas (14) |
| 40 | Personal interest | Personal Interest / Charm of the field (1)  Personal interest (2)  Personal interest/ Pursuit of non-medical interests (3)  Personal interest (5)  Personal interest (6)  Personal interest (9)  Personal interest (11)  Interesting (12)  Dedication to the field (17)  Self-interest (19)  Self-interest (21)  Interest in the specialty patient type (22)  Personal interest (24)  Interest (25)  Personal interest (27)  Personal interest (28)  Personal interest (29) |
| 41 | Mentor Influence | Mentor effect (27) |
| 42 | On-call/emergency schedules | On-call schedule/ Focus on urgent care curriculum (10)  Avoid on-calls (11)  Desire to do ambulatory care (15)  Convenient on-call schedule (21)  No on call/emergency schedule (25) |
| 43 | Work-family compatibility | Commitment to family (5)  Family responsibilities(14)  Possibility of raising a family (22)  Ease of raising a family (26)  Social and matrimonial considerations (29) |
| 44 | Competitiveness | Less competitive field (24)  Less competition (25)  Ease of entry into residency (26) |
| 45 | Social responsibility | Burden of disease /Few specialists in the country (5)  Not enough field specialists (15)  Shortage of specialist (24)  Shortage of specialists in that field (27) |
| 46 | Personal and leisure time | Having time for spouse and family/ Having time for other personal interests (2)  Sufficient time for hobbies and personal interests(14)  Sufficient time for family/personal activities (15)  Personal time (16)  The expectation of free time (22)  Time for other things in life (27)  Devoting more time to self and family (29) |
| 47 | Altruism | Having option to help people more (2)  Serve sick (3)  Opportunities for contribution to society (5)  Benefits to patients (6)  Benefits for the patient (9)  Helpful to the community (11)  Interest in helping people (15)  altruism (27)  Helping people to improve their health (29) |
| 48 | Personal/relatives experience of disease | Personal or family Illness (3)  Early experience (12)  Personal health (18)  Health history/ Care of the family/Health history of family member (19)  experiences in the specialty (22)  Personal experience (29) |
| 49 | Prospects for further development | Availability of postgraduate training (2)  Personal development(5)  Opportunities for higher studies or further specialization (14)  Keeping options open for subspecialty (15)  Possibility of studying a subspecialty (22)  Preference of terminal branch (25)  Further training after residency (26)  Anticipated future mastering skills and development (28) |
| 50 | Financial dependency status | Family support during the specialty (22) |
| 51 | Availability of specialty courses | Availability of training slots(1)  Best available course (3)  Availability of residency program locally/Availability of residency program internationally/Ease of enrollment in residency program locally/Ease of enrollment in residency program internationally (15)  Specialty not currently offered (27) |
| 52 | Future life stability | Quality of life (12) |
| 53 | School type | Type of collage (Public, Private) (23) |
| 54 | Family socioeconomic status | Family income/ Family occupation (19)  Family background (25) |
| 55 | Career features | Work satisfaction (3)  Desired practice setting (5)  Self-satisfaction (19) |
| 56 | Personal awareness of the specialty | Books/Films (3)  Awareness of time and work before choosing (18) |
| 57 | Concern about malpractice consequences | Malpractice insurance costs (15) |
| 58 | Specialty characteristics | Content of specialty (5)  Interaction with physicians (10) |

**Thematic Analysis (Categories, sub-themes, & themes)**

| **Themes** | **Sub-Themes** | **No** | **Categories** |
| --- | --- | --- | --- |
| Interpersonal effects | Encouragement and advice | 6 | Family advice |
|  |  | 7 | Teachers’ advice |
|  |  | 8 | Senior students’/doctors’ advice |
|  |  | 9 | Friends’ advice |
|  |  | 10 | Advice from others |
|  |  | 41 | Mentor influence |
|  |  | 11 | Society expectations |
|  | Effect of role models | 12 | Teacher/other physicians role models |
|  |  | 13 | Family/ friend role models |
| Influential career aspects | Quality of working life | 17 | Work safety |
|  |  | 26 | Prestige |
|  |  | 55 | Career features |
|  |  | 58 | Specialty characteristics |
|  | Career requirements | 27 | Diversity of patients |
|  |  | 36 | Extent of interaction with patients |
|  |  | 42 | On-call/emergency schedules |
|  |  | 34 | Intellectual challenging area |
|  | Future working conditions | 29 | Working schedules |
|  |  | 49 | Prospects for further development |
|  |  | 23 | Possibility of private working |
|  |  | 24 | Autonomy |
|  |  | 39 | Future practice location |
|  |  | 57 | Concern about malpractice consequences |
|  |  | 43 | Work-family compatibility |
| Educational factors | Specialty training aspects | 18 | Length of the training period |
|  |  | 19 | Difficulty of the training period |
|  |  | 44 | Competitiveness |
|  |  | 51 | Availability of specialty courses |
|  |  | 20 | lifestyle of residency |
|  | General training factors | 14 | Educational and clinical experience |
|  |  | 15 | Educational environment influence |
|  |  | 53 | School type |
| Life fulfillment aspects | Job prospects | 3 | Job opportunities |
|  |  | 5 | Job security |
|  | Economic concerns | 21 | Income |
|  |  | 22 | Financial incentives |
|  |  | 50 | Financial dependency status |
|  | Controllable lifestyle | 25 | Overall future lifestyle |
|  |  | 32 | Working hours |
|  |  | 52 | Future life stability |
|  |  | 46 | Personal and leisure time |
|  | Immigration opportunities | 4 | International job opportunities |
| Personal determinants | Practice orientation | 30 | Hospital orientation |
|  |  | 31 | Community orientation |
|  | Values and attitudes | 37 | Empathy |
|  |  | 47 | Altruism |
|  |  | 45 | Social responsibility |
|  | Specific personal factors | 1 | Personal competencies |
|  |  | 40 | Personal interest |
|  |  | 35 | Personality type |
|  |  | 56 | Personal awareness of the specialty |
|  |  | 48 | Personal/relatives experience of disease |
|  | Demographic features | 28 | Gender |
|  |  | 38 | Living location (Hometown) |
|  | Socioeconomic status | 54 | Family socioeconomic status |
|  | Specific career interests | 2 | Academic career prospect |
|  |  | 16 | Prospect of procedural work |
|  |  | 33 | Interest in research |
